# Characterization of Avian Influenza Virus H10–H12 Subtypes Isolated from Wild Birds in Shanghai, China from 2016 to 2019

**DOI:** 10.3390/v12101085

**Published:** 2020-09-25

**Authors:** Ling Tang, Wangjun Tang, Le Ming, Jianming Gu, Kai Qian, Xiaofang Li, Tianhou Wang, Guimei He

**Affiliations:** 1 Laboratory of Wildlife Epidemic Diseases, School of Life Sciences, East China Normal University, Shanghai 200063, China; 51181300137@stu.ecnu.edu.cn (L.T.); 51171300141@stu.ecnu.edu.cn (W.T.); 51191300133@stu.ecnu.edu.cn (L.M.); 18321525196@163.com (X.L.); thwang@bio.ecnu.edu.cn (T.W.); 2 Pudong District Forestry Station of Shanghai, Shanghai 200120, China; pdgujianming@163.com (J.G.); qiankai0923@163.com (K.Q.); 3 Institute of Eco-Chongming (IEC), East China Normal University, Shanghai 200063, China

**Keywords:** avian influenza virus, H10–H12 subtypes, phylogenetic analysis, surveillance, wild birds

## Abstract

H10, H11 and H12 (H10–H12) subtypes of the avian influenza virus (AIV) are associated with waterfowl. Although these subtypes of AIV are infrequently detected in nature, they can undergo reassortment with other AIV subtypes. Few H10–H12 subtypes of AIV have been isolated from wild birds in China. In this study, 12 AIV isolates of H10–H12 subtypes were identified via routine surveillance of wild birds in Shanghai, China from 2016 to 2019, including two H10, three H11 and seven H12 isolates. Sequence and phylogenetic analyses revealed that the genomic segments of the 12 isolates are highly diverse. These 12 isolates are closely related to those in the Eurasian lineage and share a high degree of sequence identity with those from wild birds and domestic ducks in countries in the East Asian–Australasian Flyway, including Japan, Korea, Bangladesh, Vietnam and China. However, parts of the genomic segments of two H12N2 isolates (NH112319-H12N2 and NH101807-H12N2) belong to the North American lineage, suggesting intercontinental reassortment among H12 AIVs in Eurasia and North American. To better understand the ecological and phylodynamic features of H10–H12 subtypes in wild birds, a large-scale surveillance of AIVs in wild birds is warranted.

## 1. Introduction

Avian influenza viruses (AIVs) isolated from birds can be divided into H1–H16 and N1–N9 subtypes based on the antigenic characteristics of the two surface proteins, hemagglutinin (HA) and neuraminidase (NA) [1]. Wild birds are natural hosts of a variety of AIVs [2], especially Charadriiformes and Anseriformes, which are distributed throughout the world [3,4]. Some AIV subtypes are rather species specific, such as H5, H7 and H9 subtypes of AIVs that are endemic in poultry [5]. H4, H11 and H13 subtypes of AIV are primarily found in shorebirds and gulls [6,7]; H3 and H6 subtypes of AIV are prevalent in waterfowl [8].

H10–H12 subtypes of AIV are also waterfowl-associated, but are infrequently detected in nature; however, they can reassort with other subtypes of AIV [9]. It has been shown that H10 AIVs can infect both humans and other mammals [10,11]. The H10N4 AIV found in farmed minks in Sweden in 1984 was the first instance of H10 AIV infection in mammals [12]. The H10N7 AIV was found to cause mass death in harbor seals in Sweden in 2014 [11]. In late 2013, a novel reassorted H10N8 AIV was found to infect people in Jiangxi, China [13]. These findings indicate that H10 AIVs can infect a wide range of hosts. Unlike H10 AIVs, H11 AIVs have not been found to infect humans; however, they may function as gene donors for other subtypes. For example, investigations of human infections by the H7N9 AIV revealed that the H11N9 AIV was the donor of its NA gene [14]. The H12 subtype of AIV was first found in Canada in 1983 in Ring-Billed Gulls [15] and was detected in Green-Winged Teals in Japan in 2015 [16]. As little research has been conducted on H12 AIVs [7,17], their ecology and phylogeny remain largely unknown.

Waterfowl play an important role in AIV transmission because AIVs in the feces of infected birds are excreted into water and can be transmitted by migratory birds to other birds and mammals [18,19]. Shanghai is a city in the Yangtze River Estuary in the East Asian–Australasian Flyway and is an important stopover and wintering site for migratory birds [20,21]. During our routine surveillance of wild birds in 2016–2019, we detected a small number of H10–H12 subtypes of AIV in this region. To expand our understanding of the ecological distribution and evolution of these rare AIV subtypes, we characterized these isolates.

## 2. Materials and Methods

### 2.1. Sample Collection

With the permission and supervision of the Shanghai Wildlife Protection and Management Office, a total of 6944 throat and anal swab samples were collected from wild birds in the Nanhui Dongtan wetland (30°51′ to 31°06′ N, 121°50′ to 121°51′ E) and the Jiuduansha Natural Reservation Zone (31°06′ to 31°14′ N, 121°46′ to 122°15′ E) in Shanghai, China from 2016 to 2019. All wild birds were released after sample collection. Each swab was placed in 2 mL of viral transport medium (VTM) composed of Hank’s balanced salt solution (pH 7.4), amphotericin B (15 µg/mL), penicillin G (100 units/mL), streptomycin (50 µg/mL) and bovine serum albumin (1%) and stored at −80 °C until used.

### 2.2. Virus Identification and Genome Sequencing

All experiments were conducted under biosafety level (BSL)-2 conditions. The swab-containing tubes were swirled, and the supernatants were collected after centrifugation. AIV RNAs were extracted using the MagMAX™ Pathogen RNA/DNA Kit (Applied Biosystems, Foster City, CA, USA) with the Magmax-96 Express instrument (Applied Biosystems). After extraction, the samples were screened for the presence of AIVs by real-time reverse transcription PCR (qRT-PCR) with primers and probes (WHO, 2009) specific to the matrix gene using a 7500 real-time PCR instrument (Applied Biosystems). The positive samples were transcribed into cDNA using the Uni12 primer (5ʹ-AGC AAA AGC AGG-3ʹ) and PrimeScript™ II 1st Strand cDNA synthesis kit (Takara, Japan). AIV subtypes were determined using primers specific to HA and NA genes [19,22], and the eight segments of the H10–H12 AIV isolates were amplified using universal primers [23]. The PCR reactions consisted of 1 μL of cDNA, 1 μL each of forward and reverse primers, 12.5 μL of Taq HS Perfect Mix (Takara, Shiga, Japan) and 10.5 μL of RNAse-free water, with a final volume of 25 μL. All PCR products were sequenced by Sangon Biotech Co, Ltd (Shanghai, China) using a BigDye termination kit on an ABI 3730 sequence analyzer (Applied Biosystems, Foster City, CA, USA).

### 2.3. Sequence Analysis

The sequences obtained were spliced and analyzed using the DNAMAN 6.0 software. The sequences of related AIV strains were downloaded from NCBI and GISAID databases. The models of nucleotide evolution for each gene of these viruses were developed using the software package jModelTest version 2.1.10 (https://github.com/ddarriba/jmodeltest2) based on the Akaike information criterion (AIC). Phylogenetic trees were constructed using the maximum-likelihood (ML) method with the software package PhyML version 3.0 (http://www.atgc-montpellier.fr/phyml/) with 100 bootstrap replicates.

## 3. Results

### 3.1. Prevalence of H10–H12 AIVs in Wild Birds

**Figure 1:**
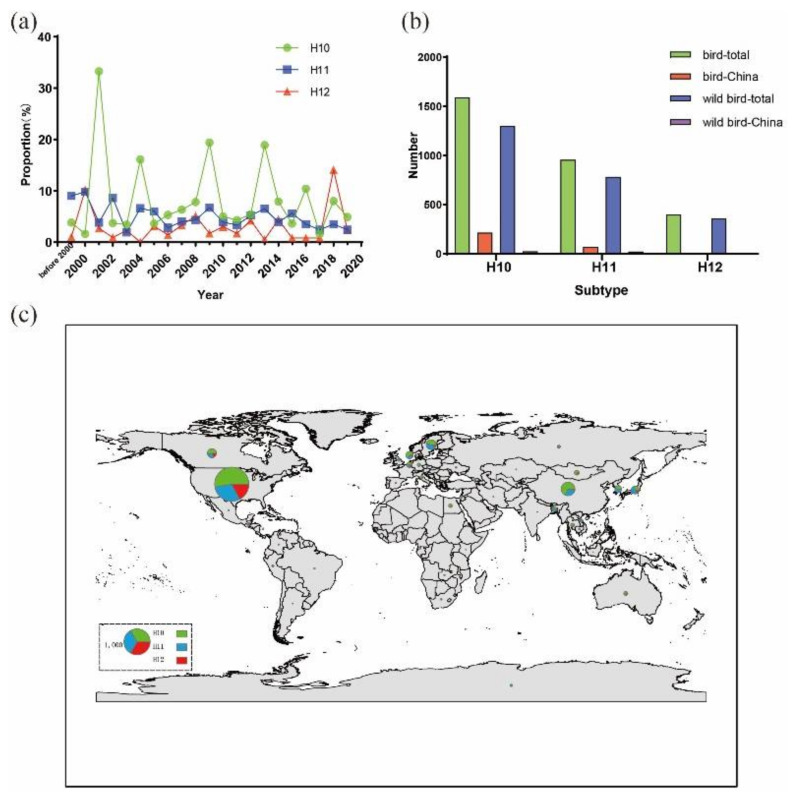
Distribution and yearly number of H10–H12 isolates. Data were obtained from the GenBank (http://www.ncbi.nlm.nih.gov) and the Global Initiative on Sharing Avian Influenza Data (www.gisaid.org) up to September 10, 2020. (**a**) Percentages of H10–H12 isolates out of the total number of AIVs per year; (**b**) total number of H10–H12 isolates from domestic and wild birds in the world and China; (**c**) global distribution of H10–H12 AIVs in wild birds. Map generated using ArcGIS version 10.2 (http://arcgis.com/). Size of each circle represents the number of isolates and the legend circle represents 1000 isolates. Green, blue and red colors represent H10, H11 and H12 isolates, respectively.

To understand the epidemiology of H10–H12 AIVs, the variation in the total number of H10–H12 isolates from 1975 to 2020 was examined using the data obtained from the GenBank (http://www.ncbi.nlm.nih.gov) and the Global Initiative on Sharing Avian Influenza Data (www.gisaid.org). After excluding duplicated data, a total of 3000 H10–H12 isolates were found up to September 10, 2020. The total number of H10–H12 isolates each year was increased since 2000, but the percentage of H10–H12 isolates out of the total number of AIVs detected per year is relatively constant. The number of H10 isolates was greater than that of H11 and H12 isolates (Figure 1a). The yearly proportion of H11 and H12 isolates was less than 10%, except for H12 isolates in 2018, which was 14.1%. For H10 isolates, the proportions were 33.3% in 2001, 16.1% in 2004, 19.4% in 2009, 18.9% in 2013, 10.4% in 2016 and less than 10% in other years, exhibiting a wave-like pattern of outbreaks with each outbreak occurring every 2–5 years. Analyses of global distribution of these isolates and their hosts revealed that more than 80% of the isolates were from wild birds (Figure 1b). Approximately 50 H10–H12 isolates found in the databases were from wild birds in China; most of them were from the wild birds in Jiangxi and Hong Kong. Only one H12 isolate was found in China (Figure 1b). These 3000 H10–H12 AIV isolates were mainly detected in North America, Europe and Asia (South Korea, Japan and China) (Figure 1c).

From 2016 to 2019, 6944 samples of throat and anal swabs from healthy wild birds in Shanghai, China were collected and 789 (11.4%) samples tested positive for AIVs by qRT-PCR. Among the positive samples, 12 H10–H12 AIV isolates were identified, including two H10, three H11 and seven H12 isolates. Of these 12 isolates, one H11 isolate was from Gruiformes and designated A/Eurasian Coot/Shanghai/PD112440/2016 (H11N9, abbreviated as PD112440-H11N9). The remaining 11 isolates were from Anseriformes and were designated A/Common Teal/Shanghai/JDS120613/2018 (JDS120613-H10N4), A/Mallard/Shanghai/JDS120662/2018 (JDS120662-H10N4), A/Common Teal/Shanghai/PD112452/2016 (PD112452-H11Nx), A/Eurasian Wigeon/Shanghai/NH101834/2017 (NH101834-H11N2), A/Common Teal/Shanghai/NH101807/2017 (NH101807-H12N2), A/Mallard/Shanghai/JDS110851/2017 (JDS110851-H12N5), A/Common Teal/Shanghai/NH102615/2018 (NH102615-H12N2), A/Common Teal/Shanghai/NH110165/2018 (NH110165-H12N2), A/Common Teal/Shanghai/NH112319/2018 (NH112319-H12N2), A/Mallard/Shanghai/NH011204/2018 (NH011204-H12N5) and A/Common Teal/Shanghai/JDS110203/2019 (JDS110203-H12N8). The full genomes of these 12 isolates were sequenced and the sequences were deposited in the GenBank. The accession numbers are shown in Table 1.

### 3.2. Molecular Characterization

The amino acid sequence motifs of the HA cleavage site were found to be ELTQGR↓GLF for JDS120613-H10N4, ELMQGR↓GLF for JDS120662-H10N4, PAIASR↓GLF for the three H11 isolates and PQAQDR↓GLF, PQAQGR↓GLF and PQVQNR↓GLF for the seven H12 isolates (Table 2), This observation indicates that all of these isolates have only one arginine at the cleavage site. The amino acids Q226 and G228 (H3 numbering) at the receptor-binding site of the HA protein are conserved among all 12 isolates, suggesting a binding preference for avian receptors [24]. The amino acid residue at position 31 of the M2 protein of all 12 isolates is serine, suggesting that they may be sensitive to M2 ion channel blockers [25]. The two amino acid residues E627 and D701 of the PB2 protein that are critical for mammalian adaption of AIVs [26,27] are also conserved throughout the 12 isolates (Table 2).

### 3.3. Sequence and Phylogenetic Analysis of H10 Isolates

Two H10N4 isolates (JDS120613-H10N4 and JDS120662-H10N4) were found in Common Teal (*Anas crecca*) and Mallard (*Anas platyrhynchos*), respectively, in the Jiuduansha Natural Reservation Zone in 2018. Except for the polymerase acidic (PA) gene of JDS120613-H10N4, the entire genomic sequences of these two isolates were determined. Results of sequence identity analysis showed that these two isolates share 92.9% to 99.3% nucleotide sequence identity among the seven genomic segments. A BLAST search (https://blast.ncbi.nlm.nih.gov/Blast.cgi) revealed that the HA genes of these two H10N4 isolates are closely related to that of A/duck/Mongolia/709/2015(H10N7) and that their NA genes are highly related to the NA genes of isolates A/duck/Mongolia/258/2011(H8N4) and A/garganey/Bangladesh/38,920/2019(H7N4). The matrix (M) gene of JDS120662-H10N4 is highly related to that of the H6 N2 AIV found in Hubei, China and the other internal genes of the two H10N4 isolates share >98.36% identity with H10, H7, H11, H8, H12 and H3 isolates found in Hokkaido, Mongolia, Egypt, Bangladesh, Georgia and Tottori (Supplementary Table S1).

**Figure 2:**
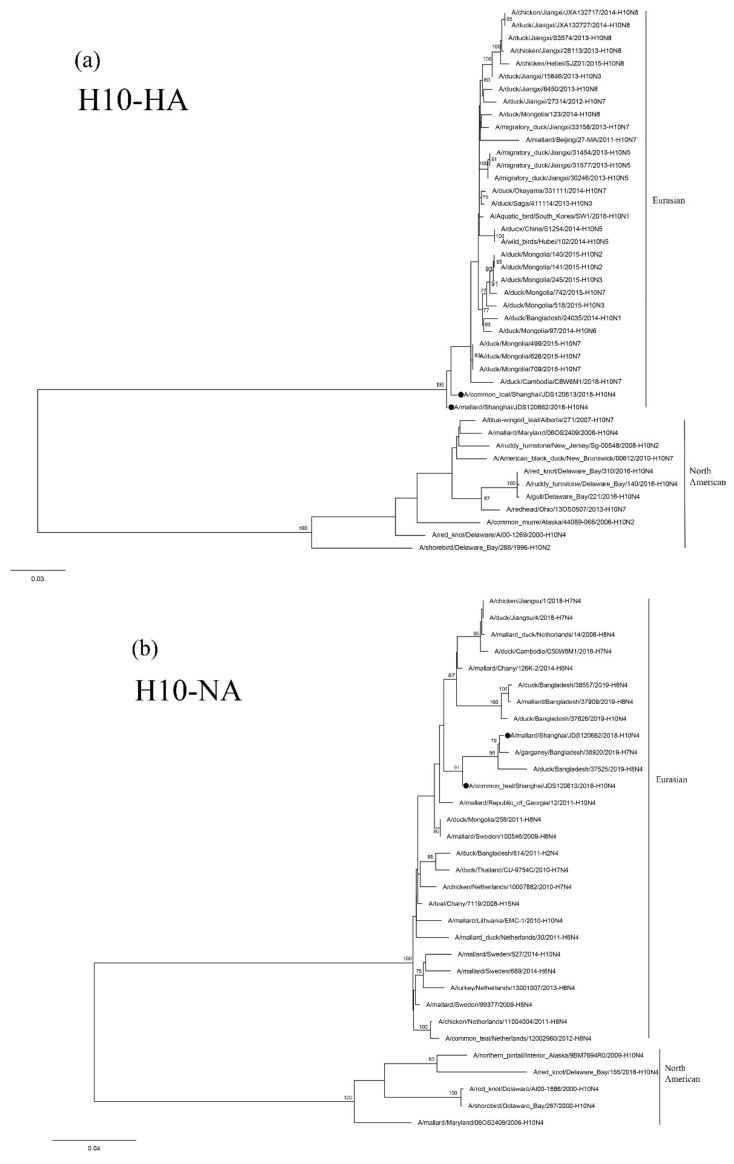
Phylogenetic trees of the HA (**a**) and NA (**b**) genes of the H10 isolates from Shanghai, China. HA and NA genes are 27–1701 bp and 1–1413 bp, respectively. Maximum-likelihood (ML) trees constructed using the GTR + G model for HA genes and the TIM2 + G model for NA genes in PhyML version 3.0. Bootstrap values were calculated for 100 replicates. Values less than 75% are not shown. Numbers indicate ML bootstrap values. H10 AIVs characterized in this study are indicated with black circles.

The HA and NA genes of isolates JDS120613-H10N4 and JDS120662-H10N4 are phylogenetically clustered in the Eurasian lineage. Their HA genes show a close relationship with those of the H10N7 isolates circulating in domestic and wild ducks in Cambodia, Mongolia, Japan and China (Figure 2a). The NA genes of these two isolates are related to those of H7N4 and H8N4 AIVs found in the Garganey and ducks in Bangladesh (Figure 2b). The PB1 polymerase (PB1) and M genes of the two H10N4 isolates are grouped together in a small sublineage and are closely related to H3 N2, H5 N2 and H6 N2 AIVs circulating in Japan and China (Supplementary Figure S1b or Supplementary Figure S1e). The nucleoprotein (NP) gene of JDS120662-H10N4 is closely related to those of the H7N7 and H7N1 AIVs found in Tottori and South Korea and that of JDS120613-H10N4 is clustered with the H5N8 AIV found in Egypt (Supplementary Figure S1d). The nonstructural (NS) genes of these two H10N4 isolates are closely related to those of H4N6 AIVs circulating in wild ducks in Tomsk and Mongolia (Supplementary Figure S1f). The PB2 polymerase (PB2) genes of these two H10N4 isolates are clustered in two different groups and are related to the AIVs isolated from ducks circulating in Tomsk, Mongolia, Japan, Bangladesh and China located along the East Asian–Australasian Flyway (Supplementary Figure S1a).

### 3.4. Sequence and Phylogenetic Analysis of H11 Isolates

Of the three H11 isolates, PD112440-H11N9 and PD112452-H11Nx were found in Eurasian Coot (*Fulica atra*) and Common Teal (*Anas crecca*), respectively. The other isolate, NH101834-H11N2, was found in Eurasian Wigeon (*Anas penelope*) in the Nanhui Dongtan wetland in Pudong in 2017. Except for the NA gene of PD112452-H11Nx, the whole genomic sequences of these three isolates were determined. The two 2016 isolates (PD112440-H11N9 and PD112452-H11Nx) are almost identical with the nucleotide sequence identity of the six genes ranging from 99.1% to 100%. In contrast, the PA genes share only 93.7% nucleotide sequence identity. Except for the NA gene, the 2017 isolate, NH101834-H11N2, share a relatively low nucleotide identity (91.0~97.6%) with the two 2016 isolates in the other seven genomic segments. These three H11 isolates exhibit a very high sequence identity with the duck and wild bird AIVs circulating in Vietnam, Japan, Korea, South Africa and China (Supplementary Table S1).

**Figure 3:**
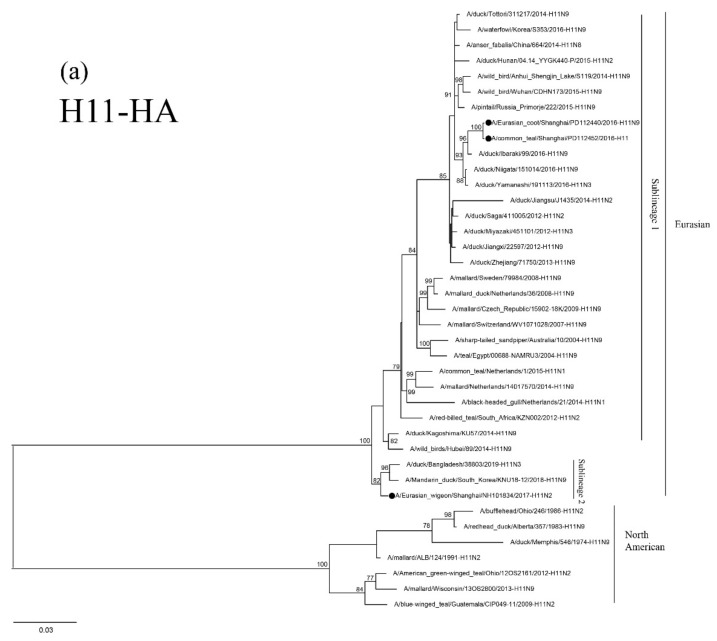

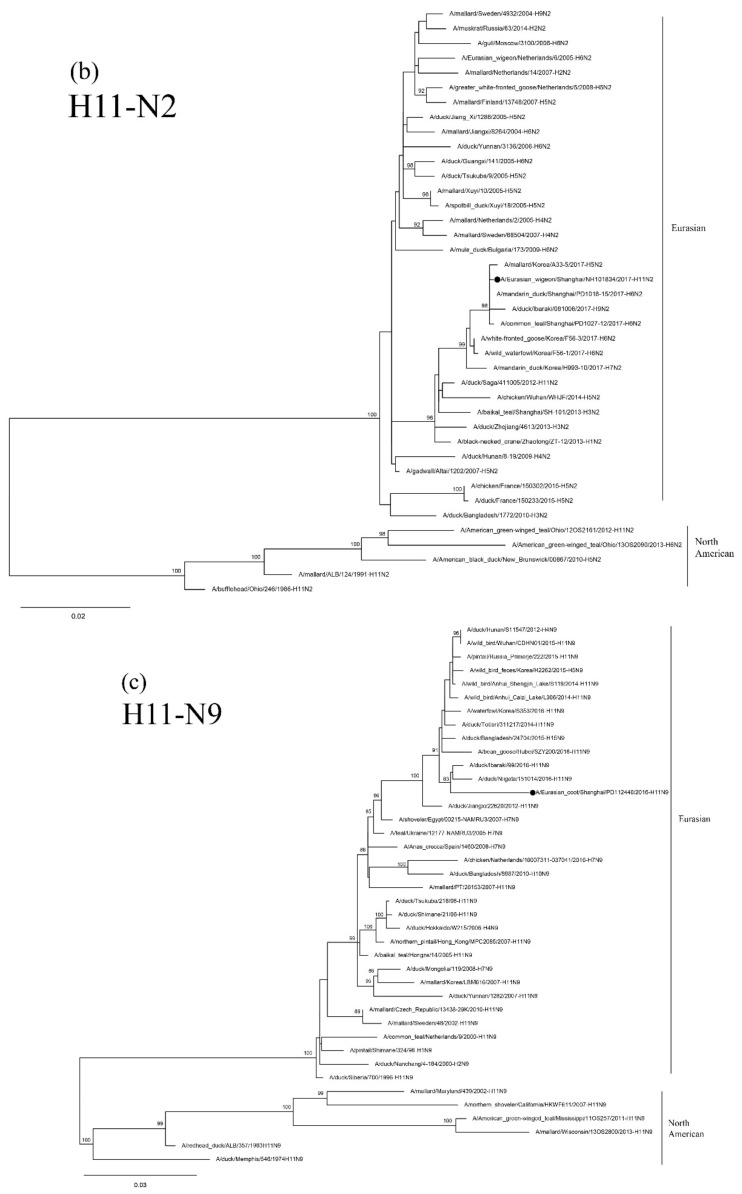
Phylogenetic trees of HA (**a**), N2 (**b**), and N9 (**c**) genes of the H11 isolates found in Shanghai, China. HA, N2 and N9 genes are 24–1688 bp, 29–1394 bp and 1–1413 bp, respectively. The maximum-likelihood (ML) trees were constructed using the TIM1+G model for HA and N2 genes and the GTR+G model for N9 genes in PhyML software version 3.0. Bootstrap values were calculated for 100 replicates. Values less than 75% are not shown. Numbers indicate ML bootstrap values. H11 AIV isolates characterized in this study are indicated with black circles.

The HA genes of the three H11 isolates are phylogenetically clustered in two Eurasian sublineages. The two 2016 isolates are grouped together in a small sublineage and are closely related to the H11N9 virus circulating in ducks in Ibaraki (A/duck/Ibaraki/99/2016). The 2017 isolate, NH101834-H11N2, is grouped in another sublineage and is closely related to H11N3 and H11N9 AIVs circulating in domestic and Mandarin Ducks in Bangladesh and South Korea (Figure 3a). The NA genes of the 2016 isolate, PD112440-H11N9, is closely related to the H11N9 AIV circulating in ducks in Japan (Figure 3c). The 2017 isolate, NH101834-H11N2, is closely related to H6 N2 and H5 N2 AIVs circulating in wild ducks in Korea and China (Figure 3b). The topologic structure of the phylogenetic tree of the six internal genes of the three isolates is similar to that of their HA gene tree (Supplementary Figure S1a–f). These three isolates are closely related to the AIVs isolated from domestic ducks and wild birds in Japan, Korea, Bangladesh, Mongolia, Vietnam, Cambodia and China.

### 3.5. Sequence and Phylogenetic Analysis of H12 Isolates

The seven H12 isolates include one H12N8, two H12N5 and four H12N2 isolates. Five of them were from Common Teal (*Anas crecca*). The other two isolates were from Mallard (*Anas platyrhynchos*) in the Jiuduansha Natural Reservation Zone and the Nanhui Dongtan wetland in Pudong. The entire genome of each of these seven H12 isolates was sequenced and analyzed. Results showed that there is a low degree of identity in the nucleotide sequences of the eight gene segments (77.9% to 99.9% for the HA gene; 51.0% to 98.8% for the NA gene; 83.7% to 96.9% for the PB2 gene; 87.9% to 99.0% for the PB1 gene; 92.1% to 99.6% for the PA gene; 92.1% to 98.9% for the NP gene and 95.0% to 98.7% for the M gene; and 70.0% to 99.7% for the NS gene). This finding indicates that these H12 isolates are highly diverse. A BLAST (https://www.ncbi.nlm.nih.gov/blast/) search showed that the HA, PB2 and PB1 genes of NH112319-H12N2 and the PB1 gene of NH101807-H12N2 share a high degree of sequence identity with those of isolates A/Mallard/Alaska/AH0029066S.1.A/2016 (H12N5) and A/Mallard/California/3070/2012 (H11N2) in the North American sublineage. The other gene segments of these H12 isolates share >97% identity with those of H12 AIVs isolated from wild birds and ducks in the East Asian–Australasian Flyway regions (e.g., Japan, Korea, Mongolia, Bangladesh, Vietnam, Netherlands and China) (Supplementary Table S1).

**Figure 4:**
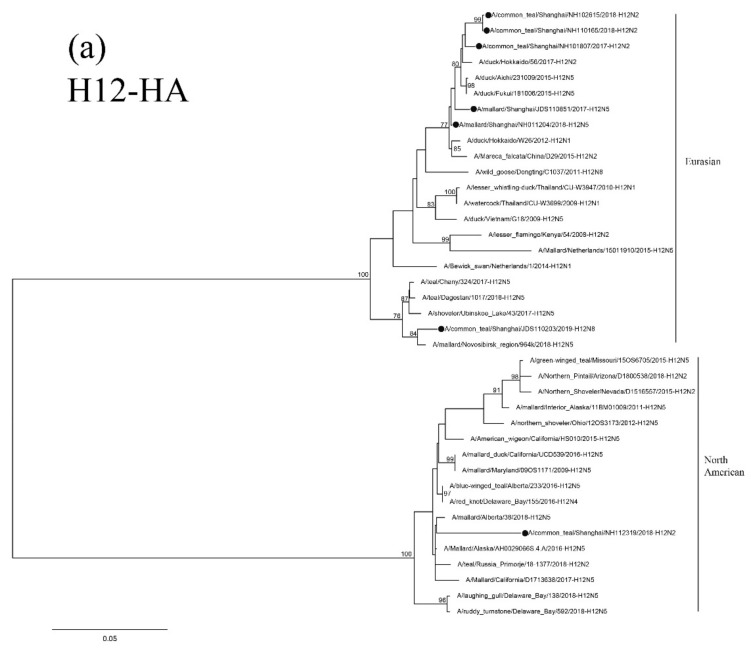

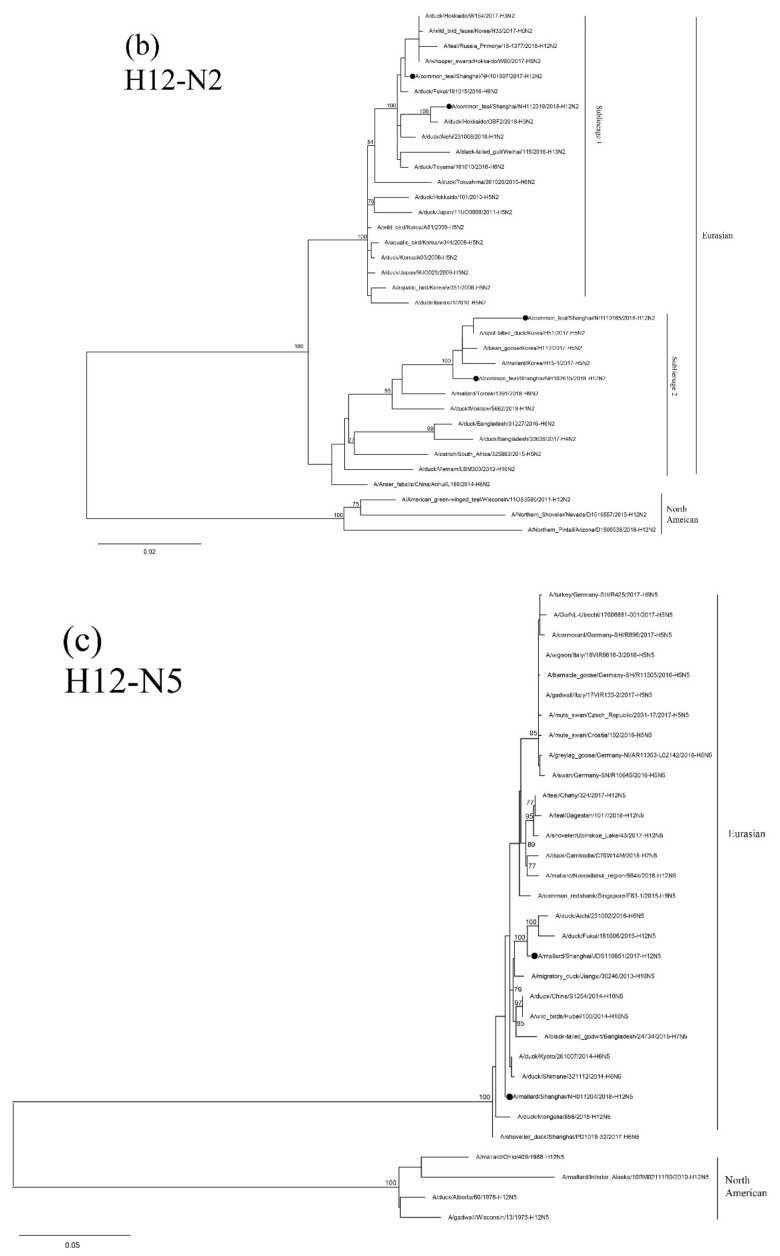

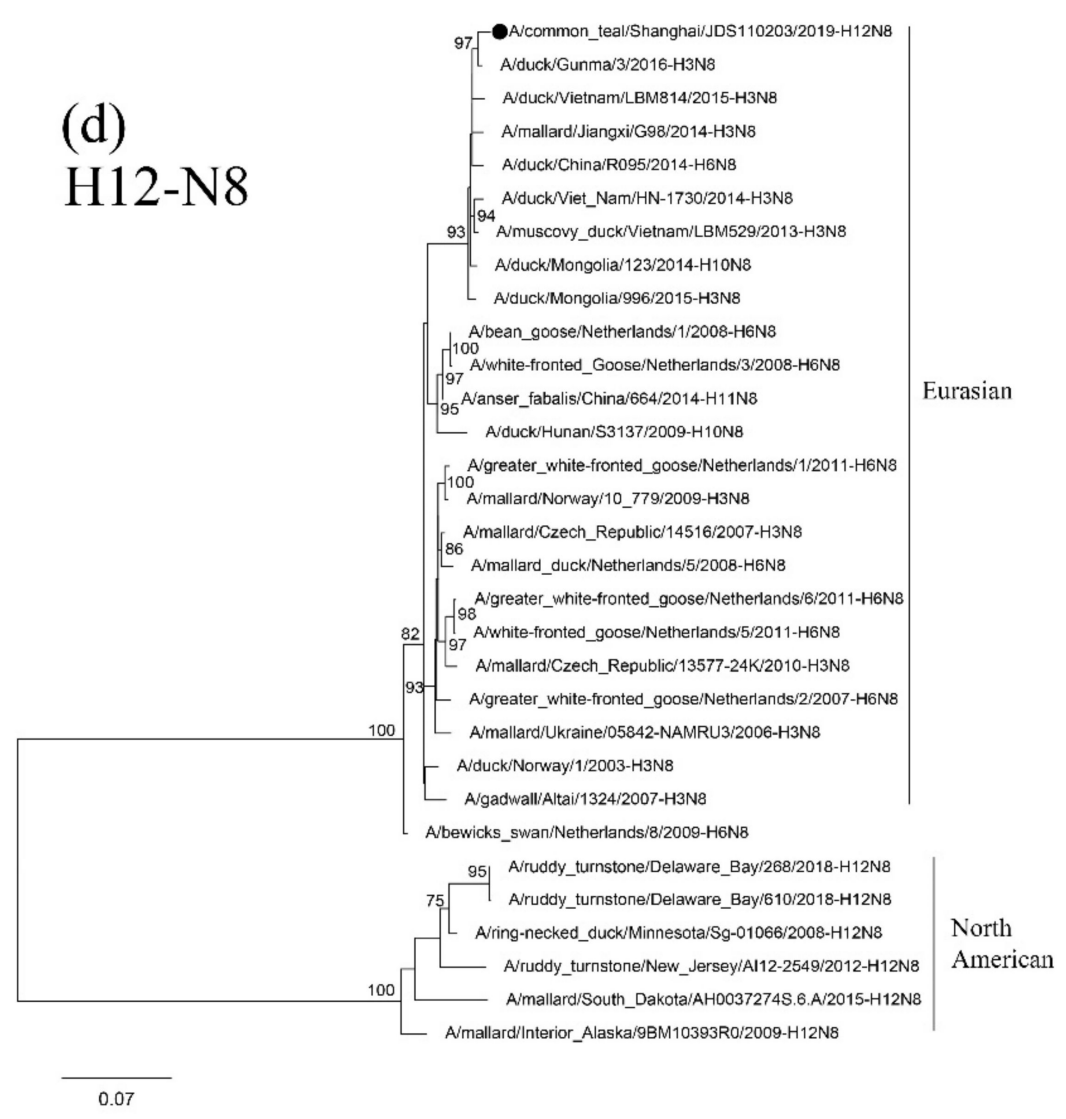
Phylogenetic trees of HA (**a**), N2 (**b**), N5 (**c**), and N8(**d**) genes of the H12 isolates found in Shanghai, China. HA, N2, N5 and N8 genes are 34–1704 bp, 24–1410 bp, 18–1399 bp and 24–1389 bp, respectively. The maximum-likelihood (ML) trees were constructed using the GTR + G model for HA, N5 and N8 genes and the TrN + I model for N2 genes in PhyML software version 3.0. Bootstrap values were calculated for 100 replicates. Values less than 75% are not shown. Numbers indicate ML bootstrap values. H12 AIV isolates characterized in this study are indicated with black circles.

Results of phylogenetic analysis showed that the seven HA genes of these H12 isolates belong to two sublineages. The isolate NH112319-H12N2 is in the North American lineage, while the other six isolates are in the Eurasian lineage (Figure 4a). Two H12N2 isolates (NH102615-H12N2 and NH110165-H12N2) are highly similar and clustered together. These two isolates are also in the same group as the isolate NH101807-H12N2 and other duck H12 AIVs found in Japan and form a sister sublineage with two H12N5 isolates. The H12N8 isolate, JDS110203-H12N8, shares a high degree of sequence identity with isolate A/Mallard/Novosibirsk region/964k/2018 (H12N5) and forms a separate clade in the Eurasian lineage.

Results of phylogenetic analysis showed that the NA genes of these H12 isolates are clustered in the Eurasian lineage. Isolates NH101807-H12N2 and NH112319-H12N2 are clustered with the AIVs found in Japan and Korea in a small sublineage. The other two H12N2 isolates and the H5 N2 isolates found in Korea form a sister sublineage (Figure 4b). The N5 and N8 genes of these H12 isolates are in the same group as those of the H6N5, H12N5 and H3N8 AIVs circulating in Mongolia, China and Japan, respectively (Figure 4c,d).

The PB2 and PB1 genes of isolate NH112319-H12N2 and the PB1 gene of isolate NH101807-H12N2 are clustered in the North American lineage. In contrast, all the other internal genes belong to the Eurasian lineage and form a separate clade with other H12 AIV isolates form migratory waterfowl, domestic aquatic birds and chickens in Japan, Korea, Mongolia, Bangladesh, Vietnam and China (Supplementary Figure S1a–f). These results suggest that the two H12N2 isolates (NH112319-H12N2 and NH101807-H12N2) were generated through genetic reassortment among viruses in Eurasian and North American virus lineages.

### 3.6. Reassortment Relationships of H10–H12 Isolates

**Figure 5:**
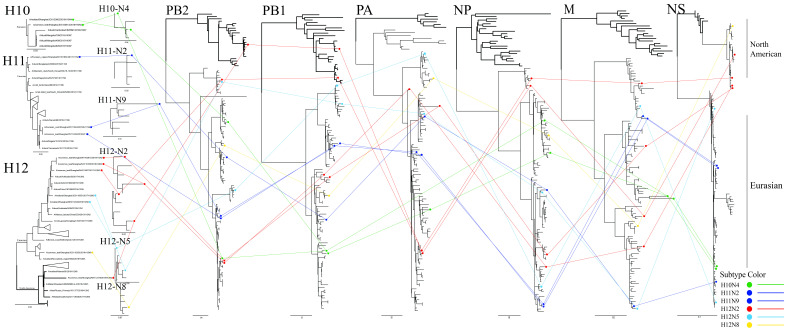
Genomic reassortment of H10–H12 isolates. ML trees contain all sequences generated in this study. Isolates and connecting lines are colored: H10—green; H11—dark blue; H12N2—red; H12N5—light blue; and H12N8—yellow. North American branches are shown in bold.

Based on the results of phylogenetic analyses of the H10–H12 isolates, the reassortment relationships of these viruses were evaluated by tracing the phylogenetic positions of their genomic segments in the phylogenetic trees. In this analysis, if the lines of the same color linking the same subtypes are parallel each other, the isolates are considered as having the same phylogenetic position, whereas if the line cross each other, the isolates are considered as having different genomic compositions. As shown in Figure 5, the two 2016 H11 isolates (dark blue lines) have the same phylogenetic positions in the eight genomic segments and are clustered together, while the 2017 H11 isolate (dark blue line) has a different phylogenetic position from the two 2016 isolates. Similarly, the two H10N4 isolates (green lines) have different phylogenetic positions in NA, PB2 and NP genes and are clustered in different groups. The H12 isolates are relatively complex with high levels of reassortment. The lines of H12N8 (yellow line), H12N5 (light blue lines) and H12N2 (red lines) of most gene segments are cross-linked, indicating different phylogenetic positions in different sublineages, while the PB2 and PB1 segments of H12N2 isolates (red lines) are closely related to those of North American isolates, indicating diverse genomic compositions.

## 4. Discussion

The increased awareness that wild birds are the natural hosts of AIVs [28] has promoted monitoring of AIVs in wild birds. Shanghai is one of the overwintering and stopover sites along the East Asian–Australasian migration route for wild birds [20,21]. Each year, millions of migratory birds of various species pass through Shanghai. The Nanhui Dongtan wetlands and the Jiuduansha Natural Reservation Zone are two major stopover sites for waterfowl in Shanghai. Although H10–H12 subtypes of AIV are detected at a very low frequency every year, they are important members of the influenza virus ecosystem and should not be ignored.

Among the twelve H10–H12 isolates (two H10, three H11 and seven H12 isolates) investigated in this study, 11 isolates were from Anseriformes, suggesting that they are major reservoirs of these rare AIV subtypes [9]. The total number of H10 isolates from birds recorded in the database is higher than that of H11 and H12 isolates (Figure 1b). This is likely due to the fact that H10N8 has caused fatal infections in humans and thus H10 AIVs have gained a greater attention in research. H12 AIVs are infrequently detected in wild birds. The NA gene of H12 AIVs is usually N2 or N5 with N2 being predominant [9]. In this study, we found one additional NA-type, N8. Among the seven H12 isolates investigated, four had the N2 gene, two had the N5 gene and one had the N8 gene. Our investigation of global distribution of H10 AIVs revealed that the occurrence frequency of H10 AIVs subtypes exhibits a wave-like pattern of outbreaks (Figure 1a); this finding is consistent with that of a long-term AIV surveillance conducted in Sweden [7]. As only two H10 isolates were found in this study, a long-term surveillance is warranted to determine whether the occurrence of H10 AIVs in Shanghai follows the global trend.

It is well known that mutations in HA and NA genes—or reassortment of genomic fragments of influenza virus—can cause the virus to escape host immunity, leading to a large-scale outbreak [29]. Some key amino acid residues, such as Q226 and G288 of the HA gene, E627 and D701 of the PB2 gene and S31 of the M2 gene are found to be conserved in all 12 isolates investigated in this study (Table 2). It is unknown whether these amino acid residues are related to the pathogenicity of AIVs in mammals, as other amino acid residues have be shown to also contribute to the virulence of AIVs [30]. Previous studies found that the change in amino acids residues related to the pathogenicity of AIVs may occurred rapidly making them able to cross species barrier and infect humans and other mammalian hosts [31]. The H10 subtype of AIV has been shown to be associated with all possible NA subtypes, thus contributing to its diversity and broad host ranges with the possibility of cross-species transmission [9]. It has been shown that H11N9 AIVs can directly transmit from ducks to humans [32]. One study found that ducks infected with the low-pathogenic H11 AIVs have a different type of immune response from those infected with other subtypes of AIV, probably due to different selective pressures [33]. As H11 AIVs may reassort with the highly pathogenic H7 AIVs [34], it is important to investigate the virulence of these rare subtypes of AIVs.

Results of phylogenetic and reassortment analysis of the H10–H12 isolates suggest that reassortment is a frequent occurrence among these isolates. For example, the two H10N4 isolates were found in birds at the same location in Jiuduansha Natural Reservation Zone in 2018, but parts of their internal gene segments belong to different sublineages (Supplementary Figure S1). This finding suggests that these two H10N4 isolates were derived from different ancestors. Similar situations are found in H12N2 and H12N5 isolates. Wild birds rather than poultry have been shown to be the main hosts for AIV reassortment [35]. Several studies have shown that intercontinental transmission of highly pathogenic AIVs (e.g., H5N8 viruses) by migratory birds frequently occurs [36,37,38]; however, it is rare for low-pathogenic AIVs to be transmitted by wild birds [39,40,41]. It has been shown that the spread of Eurasian AIVs to North America by migratory birds is common, but the transmission of America AIVs to Asia is exceedingly rare [37,38]. In this study, intercontinental reassortment of AIVs between Eurasian and North American strains was observed in the two H12N2 isolates (NH112319-H12N2 and NH101807-H12N2).

Results of genetic analyses of the 12 H10–H12 isolates revealed that there is a high degree of diversity of the AIVs circulating in wild birds. This situation may pose a threat to public health due to the frequent reassortment among wild birds and poultry. Because Shanghai is an important ecological site for AIVs in China and around the world, monitoring and studying the rare AIV subtypes in this region is of great importance. In the future, AIV surveillance in the migratory flyways should be strengthened to provide a timely and effective strategy to control AIV infections throughout the world.

## Figures and Tables

**Table 1:** Twelve H10–H12 isolates and GenBank accession numbers of their genomic sequences.

Isolates	Abbreviation	Subtype	Accession Numbers in GenBank
A/Common Teal/Shanghai/JDS120613/2018(H10N4)	JDS120613-H10N4	H10N4	MN049531–MN049537
A/Mallard/Shanghai/JDS120662/2018(H10N4)	JDS120662-H10N4	H10N4	MN049523–MN049530
A/Eurasian Coot/Shanghai/PD112440/2016(H11N9)	PD112440-H11N9	H11N9	MN049550–MN059557
A/Common Teal/Shanghai/PD112452/2016(H11Nx)	PD112452-H11Nx	H11Nx	MN044998–MN045004
A/Eurasian Wigeon/Shanghai/NH101834/2017(H11N2)	NH101834-H11N2	H11N2	MN044910–MN044917
A/Common Teal/Shanghai/NH101807/2017(H12N2)	NH101807-H12N2	H12N2	MN049563–MN049569
A/Mallard/Shanghai/JDS110851/2017(H12N5)	JDS110851-H12N5	H12N5	MN049575–MN049581
A/Common Teal/Shanghai/NH102615/2018(H12N2)	NH102615-H12N2	H12N2	MN121558–MN121565
A/Common Teal/Shanghai/NH110165/2018(H12N2)	NH110165-H12N2	H12N2	MN122300–MN122307
A/Common Teal/Shanghai/NH112319/2018(H12N2)	NH112319-H12N2	H12N2	MN049593–MN049600
A/Mallard/Shanghai/NH011204/2018(H12N5)	NH011204-H12N5	H12N5	MN049584–MN049591
A/Common Teal/Shanghai/JDS110203/2019(H12N8)	JDS110203-H12N8	H12N8	MN795764–MN795771

**Table 2:** Molecular characteristics of the 12 H10–H12 isolates.

Virus	Amino Acid Sequence at HA Cleavage Site	HA Receptor-Binding Site		M2 Key Site	PB2	
		226	228	31	627	701
JDS120613-H10N4	ELTQGR↓GLF	Q	G	S	E	D
JDS120662-H10N4	ELMQGR↓GLF	Q	G	S	E	D
PD112440-H11N9	PAIASR↓GLF	Q	G	S	E	D
PD112452-H11Nx	PAIASR↓GLF	Q	G	S	E	D
NH101834-H11N2	PAIASR↓GLF	Q	G	S	E	D
NH101807-H12N2	PQAQDR↓GLF	Q	G	S	E	D
JDS110851-H12N5	PQAQDR↓GLF	Q	G	S	E	D
NH102615-H12N2	PQAQDR↓GLF	Q	G	S	E	D
NH110165-H12N2	PQAQDR↓GLF	Q	G	S	E	D
NH112319-H12N2	PQAQGR↓GLF	Q	G	S	E	D
NH011204-H12N5	PQAQDR↓GLF	Q	G	S	E	D
JDS110203-H12N8	PQVQNR↓GLF	Q	G	S	E	D

## Data Availability

All data generated in this study are included in this article.

## Supplementary Materials

The following are available online at https://www.mdpi.com/1999-4915/12/10/1085/s1, Figure S1: Phylogenetic trees of the internal genes of the H10-H12 subtype isolates found in Shanghai, China, Table S1: Viruses in the GenBank database exhibiting the highest homology with the H10-H12 subtype isolates in this study.
