# Supplementary material for: Characterization of Avian Influenza Virus H10–H12 Subtypes Isolated from Wild Birds in Shanghai, China from 2016 to 2019
Source: Viruses. 2020 Sep 25;12(10):1085. doi: 10.3390/v12101085 (PMC7600165; doi:10.3390/v12101085)
Supplement: Supplementary file 1 [file viruses-12-01085-s001.zip › supplementary Figure S1.docx]

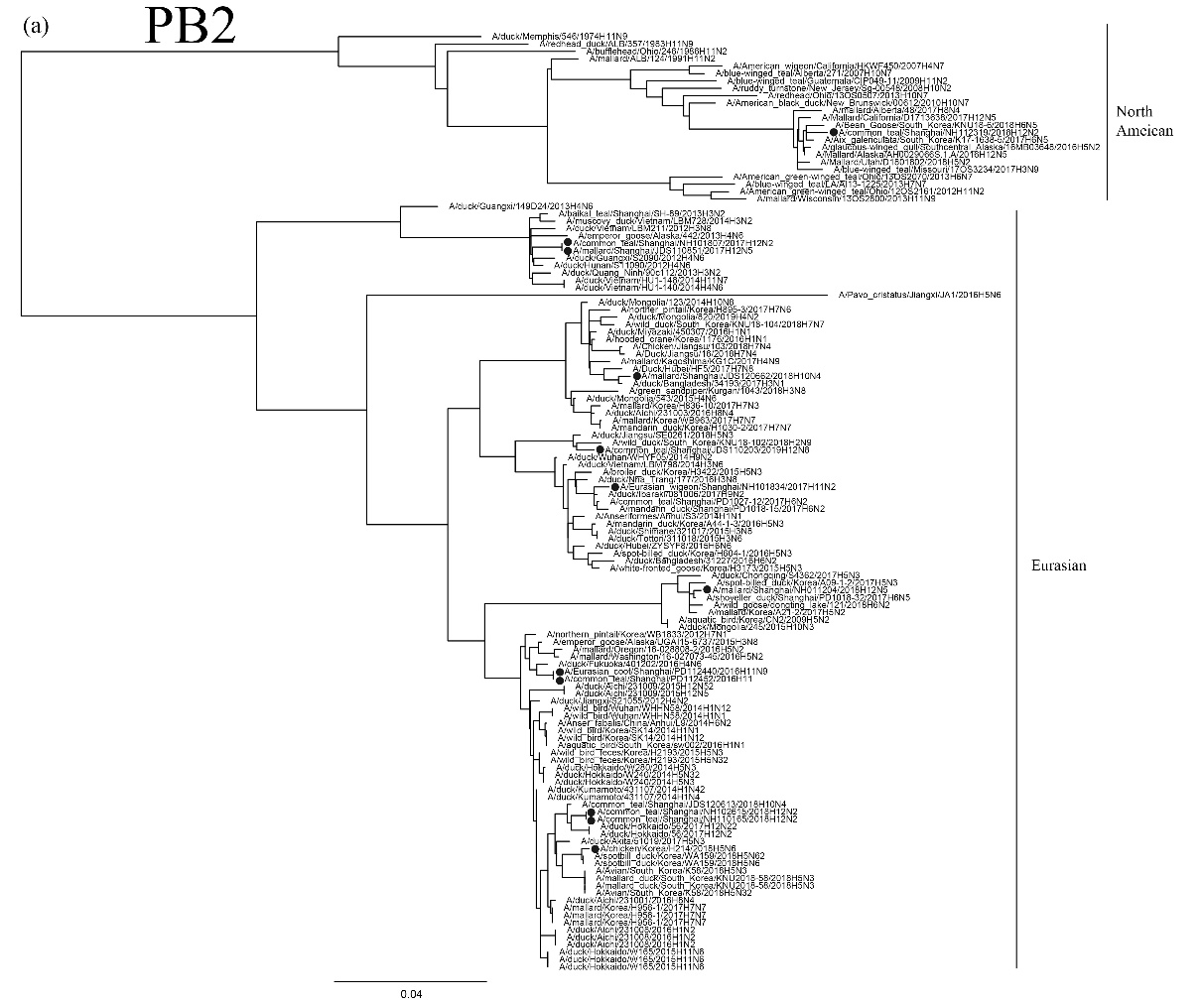


**Supplementary Figure S1a.** Phylogenetic trees of the PB2 genes of the H10-H12 subtype isolates found in Shanghai, China. PB2 genes are 21-2303 bp. The maximum likelihood (ML) tree was constructed using the GTR+G model in PhyML software version 3.0. Bootstrap values were calculated for 1,000 replicates, and values less than 75% are not shown. Number indicate the ML bootstrap values. The viruses characterized in this study are indicated by black circles.


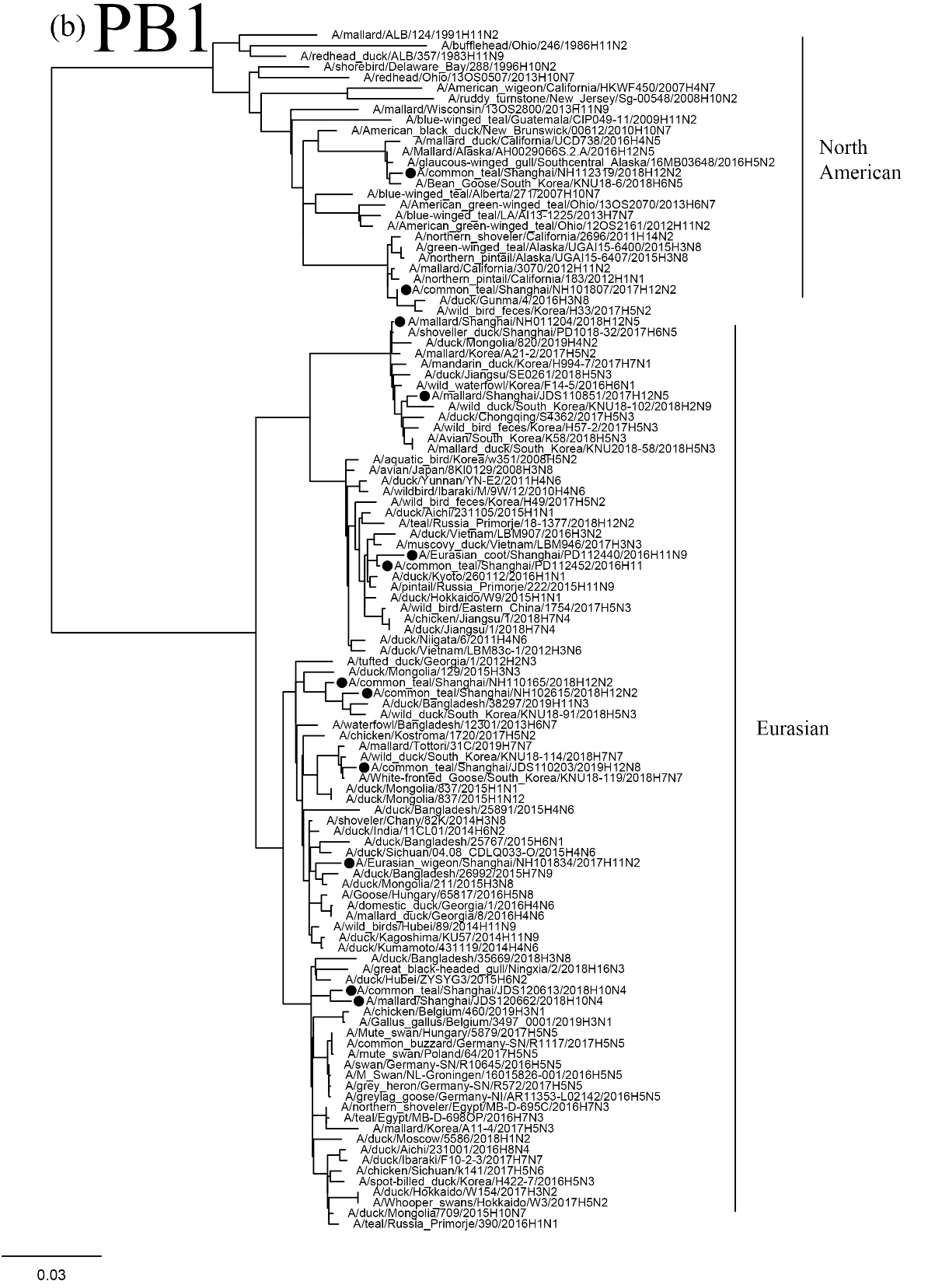


**Supplementary Figure S1b.** Phylogenetic trees of the PB1 genes of the H10-H12 subtype strains isolated in Shanghai, China. PB1 genes are 18-2291 bp. The analysis methods were the same as described in Figure S1a.


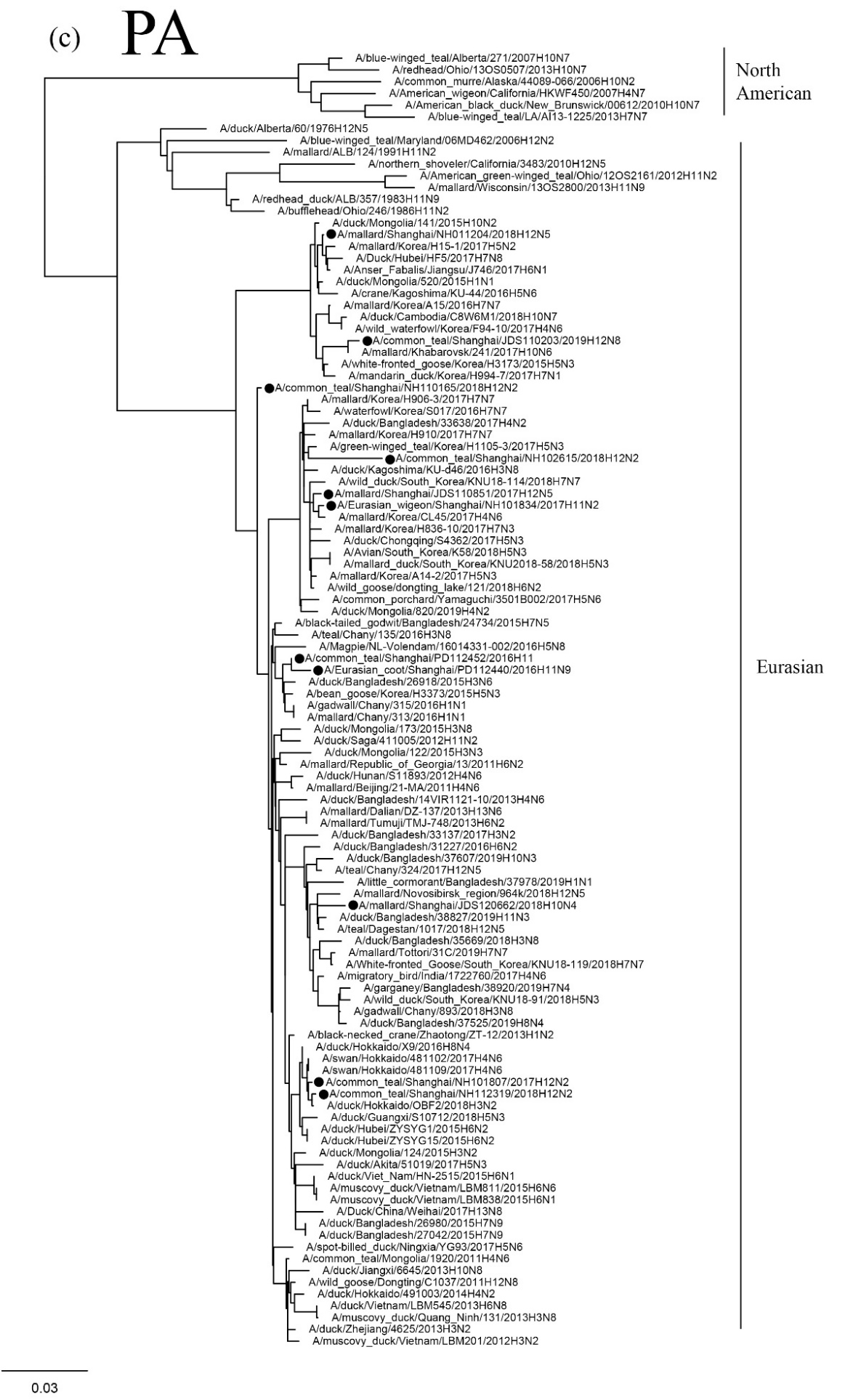


**Supplementary Figure S1c.** Phylogenetic trees of the PA genes of the H10-H12 subtype strains isolated in Shanghai, China. PA genes are 18-2167 bp. The analysis methods were the same as described in Figure S1a.


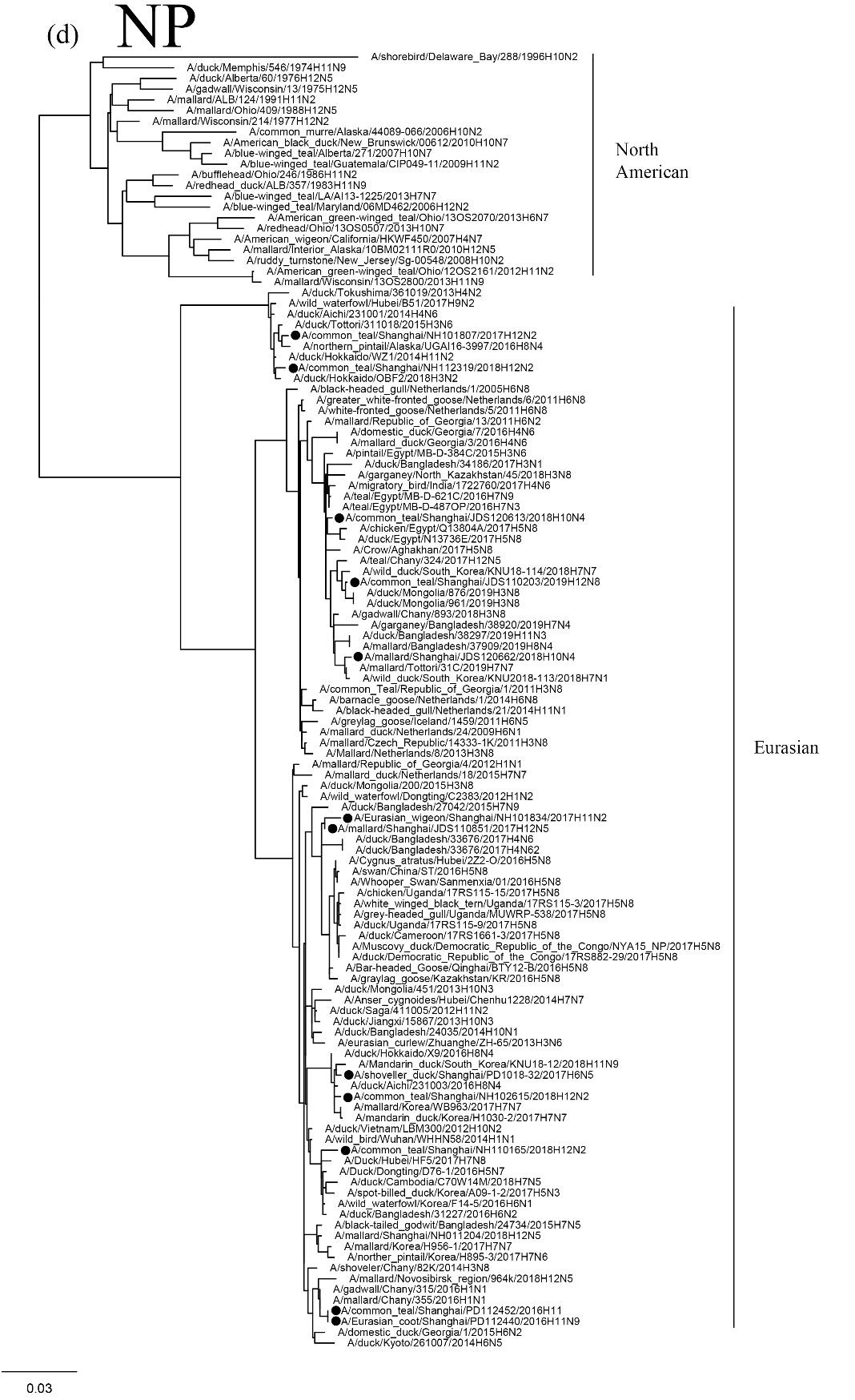


**Supplementary Figure S1d.** Phylogenetic trees of the NP genes of the H10-H12 subtype strains isolated in Shanghai, China. PB2 genes are 46-1543 bp. The maximum likelihood (ML) tree was constructed using the GTR+I+G model in PhyML software version 3.0. Bootstrap values were calculated for 1,000 replicates, and values less than 75% are not shown. Number indicate the ML bootstrap values. The viruses characterized in this study are indicated by black circles.


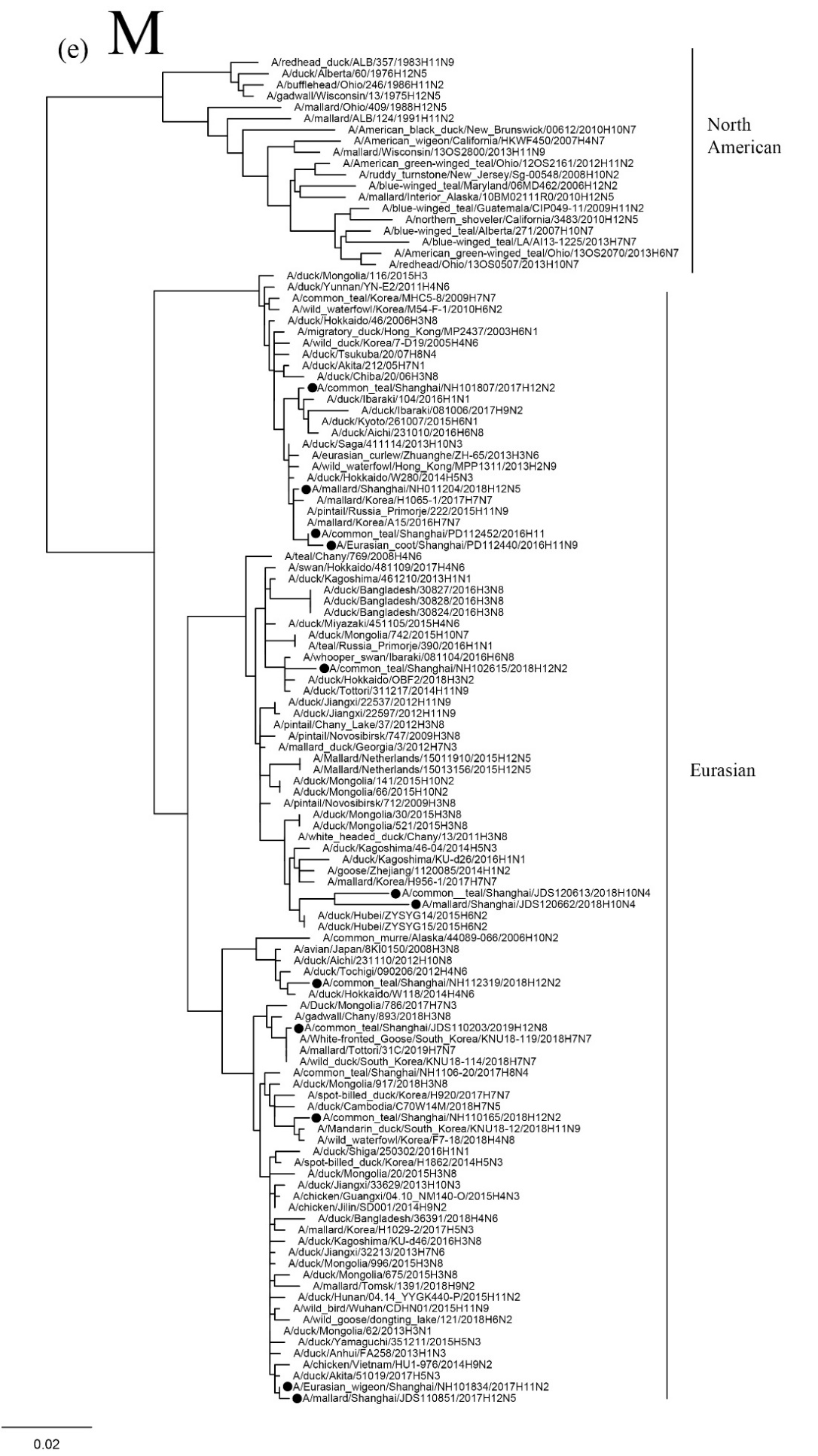


**Supplementary Figure S1e.** Phylogenetic trees of the M genes of the H10-H12 subtype strains isolated in Shanghai, China. M genes are 19-1000 bp. The analysis methods were the same as described in Figure S1d.


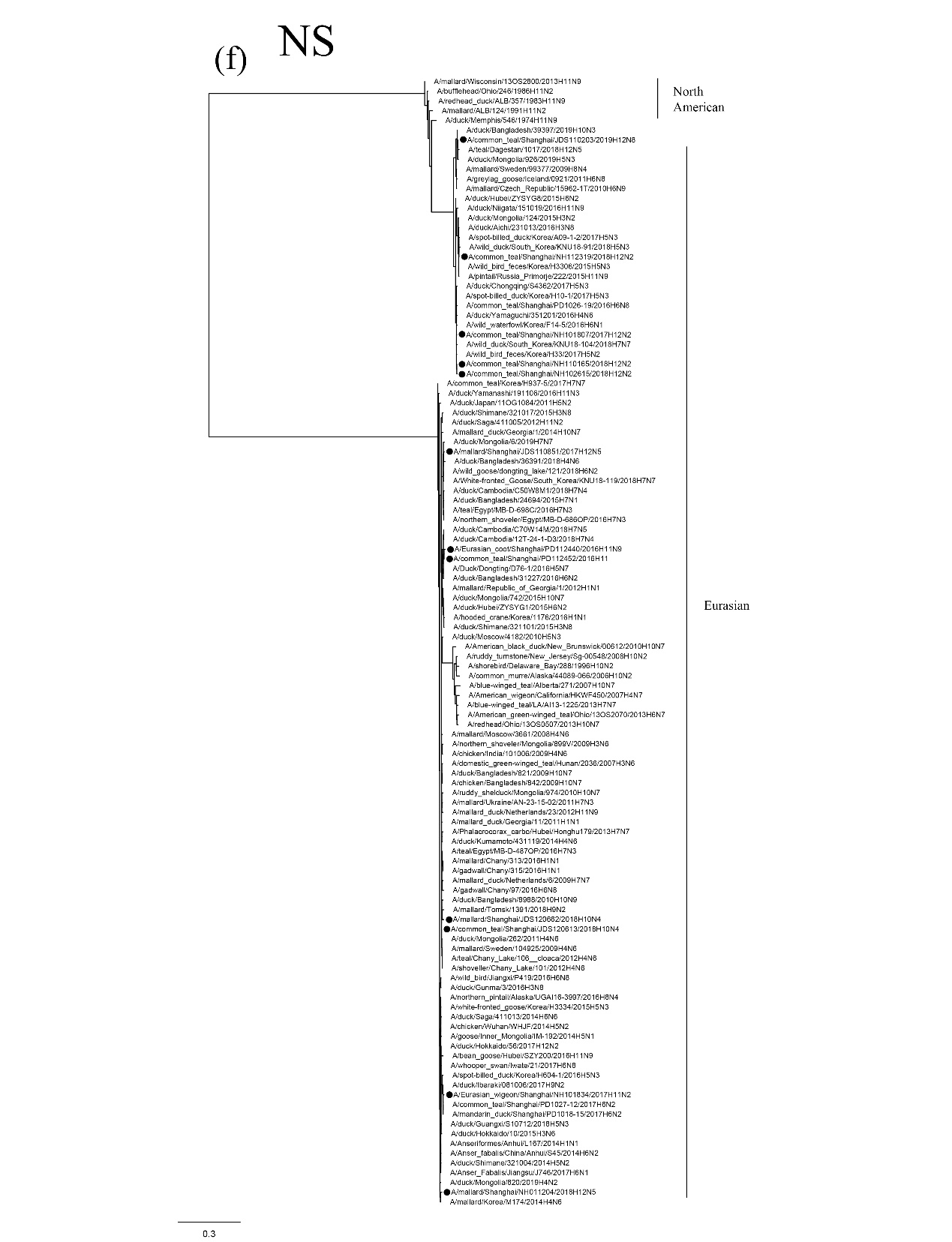


**Supplementary Figure S1f.** Phylogenetic trees of the NS genes of the H10-H12 subtype strains isolated in Shanghai, China. NS genes are 1-838 bp. The maximum likelihood (ML) tree was constructed using the TPM2uf+I+G model in PhyML software version 3.0. Bootstrap values were calculated for 1,000 replicates, and values less than 75% are not shown. Number indicate the ML bootstrap values. The viruses characterized in this study are indicated by black circles.
