# Supplementary material for: Characterization of Avian Influenza Virus H10–H12 Subtypes Isolated from Wild Birds in Shanghai, China from 2016 to 2019
Source: Viruses. 2020 Sep 25;12(10):1085. doi: 10.3390/v12101085 (PMC7600165; doi:10.3390/v12101085)
Supplement: Supplementary file 1 [file viruses-12-01085-s001.zip › supplementary Table S1.docx]

**Supplementary Table S1.** Viruses in the GenBank database exhibiting the highest homology with the H10-H12 subtype isolates in this study

| **Virus name** | **Gene** | **The most homologous strain** | **Identity** |
| --- | --- | --- | --- |
| A/common teal/Shanghai/JDS120613/2018(H10N4) | PB2 | A/duck/Hokkaido/56/2017(H12N2) | 99.25% |
|  | PB1 | A/duck/Mongolia/709/2015(H10N7) | 99.21% |
|  | HA | A/duck/Mongolia/709/2015(H10N7) | 98.58% |
|  | NP | A/teal/Egypt/MB-D-487OP/2016(H7N3) | 99.60% |
|  | NA | A/duck/Mongolia/258/2011(H8N4) | 98.62% |
|  | M | A/mallard duck/Georgia/3/2012(H7N3) | 98.37% |
|  | NS | A/duck/Bangladesh/821/2009(H10N7) | 99.77% |
| A/mallard/Shanghai/JDS120662/2018(H10N4) | PB2 | A/duck/Bangladesh/34193/2017(H3N1) | 99.51% |
|  | PB1 | A/northern shoveler/Egypt/MB-D-695C/2016(H7N3) | 98.36% |
|  | PA | A/duck/Bangladesh/38827/2019(H11N3) | 98.67% |
|  | HA | A/duck/Mongolia/709/2015(H10N7) | 98.52% |
|  | NP | A/mallard/Tottori/31C/2019(H7N7) | 99.60% |
|  | NA | A/garganey/Bangladesh/38920/2019(H7N4) | 99.29% |
|  | M | A/duck/Hubei/ZYSYG15/2015(H6N2) | 98.50% |
|  | NS | A/teal/Egypt/MB-D-487OP/2016(H7N3) | 98.96% |
| A/Eurasian coot/Shanghai/PD112440/2016(H11N9)  A/common teal/Shanghai/PD112452/2016(H11Nx)  A/Eurasian wigeon/Shanghai/NH101834/2017(H11N2)  A/common teal/Shanghai/NH101807/2017(H12N2)  A/mallard/Shanghai/JDS110851/2017(H12N5)  A/common teal/Shanghai/NH102615/2018(H12N2)  A/common teal/Shanghai/NH110165/2018(H12N2)  A/common teal/Shanghai/NH112319/2018(H12N2)  A/mallard/Shanghai/NH011204/2018(H12N5)  A/common teal/Shanghai/JDS110203/2019(H12N8) | PB2  PB1  PA  HA  NP  NA  M  NS  PB2  PB1  PA  HA  NP  M  NS  PB2  PB1  PA  HA  NP  NA  M  NS  PB2  PB1  PA  HA  NP  NA  M  NS  PB2  PB1  PA  HA  NP  NA  M  NS  PB2  PB1  PA  HA  NP  NA  M  NS  PB2  PB1  PA  HA  NP  NA  M  NS  PB2  PB1  PA  HA  NP  NA  M  NS  PB2  PB1  PA  HA  NP  NA  M  NS  PB2  PB1  PA  HA  NP  NA  M  NS | A/emperor goose/Alaska/UGAI15-6737/2015(H3N8)  A/duck/Hokkaido/W9/2015(H1N1)  A/duck/Bangladesh/26918/2015(H3N6)  A/Anser fabalis/China/664/2014(H11N8)  A/domestic duck/Georgia/1/2015(H6N2)  A/duck/Bangladesh/24704/2015(H15N9)  A/duck/Hokkaido/W280/2014(H5N3)  A/Duck/Dongting/D76-1/2016(H5N7)  A/emperor goose/Alaska/UGAI15-6737/2015(H3N8)  A/duck/Hokkaido/W9/2015(H1N1)  A/duck/Bangladesh/26918/2015(H3N6)  A/Anser fabalis/China/664/2014(H11N8)  A/domestic duck/Georgia/1/2015(H6N2)  A/duck/Saga/411114/2013(H10N3)  A/Duck/Dongting/D76-1/2016(H5N7)  A/mandarin duck/Shanghai/PD1018-15/2017(H6N2)  A/duck/Mongolia/211/2015(H3N8)  A/mallard/Korea/CL45/2017(H4N6)  A/Mandarin duck/South Korea/KNU18-12/2018(H11N9)  A/Whooper Swan/Sanmenxia/01/2016(H5N8)  A/mandarin duck/Shanghai/PD1018-15/2017(H6N2)  A/duck/Akita/51019/2017(H5N3)  A/common teal/Shanghai/PD1027-12/2017(H6N2)  A/Pavo cristatus/Jiangxi/JA1/2016(H5N6)  A/mallard/California/3070/2012(H11N2)  A/swan/Hokkaido/481109/2017(H4N6)  A/duck/Hokkaido/56/2017(H12N2)  A/duck/Hokkaido/WZ1/2014(H11N2)  A/duck/Hokkaido/W154/2017(H3N2)  A/duck/Saga/411114/2013(H10N3)  A/duck/Hubei/ZYSYG8/2015(H6N2)  A/mallard/Kagoshima/KG1C/2017(H4N9)  A/mallard duck/South Korea/KNU2018-58/2018(H5N3)  A/wild goose/Dongting lake/121/2018(H6N2)  A/duck/Hokkaido/56/2017(H12N2)  A/Whooper Swan/Sanmenxia/01/2016(H5N8)  A/migratory duck/Jiangxi/30246/2013(H10N5)  A/duck/Mongolia/62/2013(H3N1)  A/wild goose/dongting lake/121/2018(H6N2)  A/duck/Hokkaido/56/2017(H12N2)  A/wild duck/South Korea/KNU18-91/2018(H5N3)  A/wild goose/dongting lake/121/2018(H6N2)  A/duck/Hokkaido/56/2017(H12N2)  A/duck/Hokkaido/X9/2016(H8N4)  A/duck/Moscow/5662/2018(H1N2)  A/duck/Hokkaido/OBF2/2018(H3N2)  A/duck/Chongqing/S4362/2017(H5N3)  A/duck/Hokkaido/W165/2015(H11N6)  A/wild duck/South Korea/KNU18-91/2018(H5N3)  A/black-tailed godwit/Bangladesh/24734/2015(H7N5)  A/duck/Hokkaido/56/2017(H12N2)  A/duck/Bangladesh/31227/2016(H6N2)  A/Anser fabalis/China/Anhui/L180/2014(H6N2)  A/Mandarin duck/South Korea/KNU18-12/2018(H11N9)  A/common teal/Shanghai/PD1026-19/2016(H6N8)  A/Mallard/Alaska/AH0029066S.1.A/2016(H12N5)  A/Mallard/Alaska/AH0029066S.2.A/2016(H12N5)  A/duck/Hokkaido/OBF2/2018(H3N2)  A/Mallard/Alaska/AH0029066S.4.A/2016(H12N5)  A/duck/Hokkaido/WZ1/2014(H11N2)  A/duck/Hokkaido/OBF2/2018(H3N2)  A/duck/Hokkaido/W118/2014(H4N6)  A/duck/Mongolia/124/2015(H3N2)  A/wild goose/dongting lake/121/2018(H6N2)  A/wild waterfowl/Korea/F14-5/2016(H6N1)  A/duck/Mongolia/520/2015(H1N1)  A/duck/Hokkaido/W26/2012(H12N1)  A/black-tailed godwit/Bangladesh/24734/2015(H7N5)  A/wild birds/Hubei/100/2014(H10N5)  A/duck/Hokkaido/W280/2014(H5N3)  A/mallard/Korea/M174/2014(H4N6)  A/duck/Jiangsu/SE0261/2018(H5N3)  A/White-fronted Goose/South Korea/KNU18-119/2018(H7N7)  A/duck/Mongolia/141/2015(H10N2)  A/Bewick swan/Netherlands/1/2014(H12N1)  A/duck/Mongolia/961/2019(H3N8)  A/muscovy duck/Vietnam/LBM529/2013(H3N8)  A/mallard/Tottori/31C/2019(H7N7)  A/duck/Mongolia/926/2019(H5N3) | 99.05%  98.72%  94.99%  98.29%  98.98%  98.57%  99.21%  99.19%  98.94%  99.12%  99.11%  98.38%  98.99%  99.56%  99.53%  99.65%  98.80%  99.53%  98.79%  98.51%  100%  99.58%  99.50%  99.91%  99.46%  99.63%  99.02%  99.33%  99.71%  99.49%  99.28%  99.51%  99.13%  99.34%  98.60%  99.18%  98.84%  99.60%  99.62%  98.94%  98.73%  97.07%  98.72%  99.59%  97.79%  99.25%  99.03%  97.52%  98.55%  98.85%  98.80%  99.12%  96.69%  99.09%  99.37%  98.58%  99.35%  99.77%  96.45%  99.46%  99.43%  99.22%  99.52%  99.25%  99.56%  99.58%  99.38%  99.60%  99.20%  99.70%  99.17%  99.39%  99.52%  98.16%  95.20%  99.54%  98.39%  99.90%  99.54% |
